# A Viral Protein Antagonist for Both AID and APOBEC3

**DOI:** 10.3390/v18040399

**Published:** 2026-03-24

**Authors:** Jaquelin P. Dudley

**Affiliations:** Department of Molecular Biosciences and LaMontagne Center for Infectious Disease, The University of Texas at Austin, Austin, TX 78712, USA; jdudley@austin.utexas.edu; Tel.: +1-(512)-471-8415

**Keywords:** innate immunity, Rem, activation-induced cytidine deaminase, viral DNA mutagenesis, APOBEC antagonist, ERAD

## Abstract

The APOBEC family of cytidine deaminases is part of the innate immune response to infections by multiple RNA- and DNA-containing viruses. Since the activity of these enzymes, typically APOBEC3, often involves mutations that inhibit or block viral replication, viruses have evolved antagonists that limit APOBEC function. The retrovirus mouse mammary tumor virus (MMTV) encodes an APOBEC antagonist, Rem. Surprisingly, Rem appears to inhibit APOBEC3 through proteasomal degradation of a different APOBEC enzyme, AID.

## 1. APOBEC Enzymes as Viral Antagonists

The innate immune response has many facets involving multiple cell types that are on the front lines of host defense against pathogens. Dendritic cells, macrophages, and natural killer (NK) cells are critical components of innate immunity as well as targets for viral infections [1]. Such cells can facilitate infections of B and T lymphocytes, which mediate adaptive immunity that provides both specificity and memory [2]. All these cell types are known to express molecules that block viral replication, including the apolipoprotein B mRNA-editing catalytic polypeptide-like (APOBEC) family of cytidine deaminases [3,4,5]. The human genome encodes 11 APOBEC enzymes: APOBEC1 (hA1), APOBEC2 (hA2), APOBEC3A (hA3A), APOBEC3B (hA3B), APOBEC3C (hA3C), APOBEC3D (hA3D), APOBEC3F (hA3F), APOBEC3G (hA3G), APOBEC3H (hA3H), APOBEC4 (hA4), and activation-induced cytidine deaminase (hAID) [3]. Each of these deaminases targets cytidines in preferred sequence contexts on single-stranded RNA and DNA [6,7,8] (reviewed in [3,5]). The mouse genome encodes APOBEC1, 2, 4 (mA1, mA2, mA4), and mAID, but only a single A3 enzyme (mA3) [3]. Recent evidence indicates that house mice have two mA3 alleles, which have different target specificities [9]. The A3 proteins are the primary viral antagonists and have been shown to block retroviral replication by inhibiting reverse transcription and/or deaminating cytidines during minus-strand DNA synthesis [10,11,12]. Cytidine deamination of the viral minus strand often leads to G-to-A substitution mutations on the plus strand, which inactivates required viral proteins [5]. Alternatively, host-encoded enzymes lead to degradation of damaged or incomplete reverse transcription products [13]. Therefore, the A3 deaminases represent a major block to viral replication in cell types likely to provide the first barrier to virus dissemination in the host.

## 2. Conceptual Overview

In this review, I present the idea that the retrovirus mouse mammary tumor virus (MMTV) has evolved to antagonize multiple APOBECs during replication in different cell types by targeting AID: the most ancient of this cytidine deaminase family. Genetic, virological, and biochemical data are consistent with a model in which the MMTV-encoded Rem protein degrades AID, which serves as an adapter and regulator of antiviral A3 enzymes.

## 3. MMTV-Encoded Rem as an APOBEC Inhibitor

Viruses, ever on the defensive to maintain their lifestyle, have evolved multiple methods to overcome APOBEC restrictions (reviewed in [3,5,14]). The first known viral APOBEC inhibitor was the Vif protein encoded by human immunodeficiency virus type 1 (HIV-1) [11]. HIV-1 strains lacking Vif expression were shown to accumulate G-to-A mutations in viral DNA, which restricted HIV-1 replication in certain T-cell lines, such as H9 and MT-2 [15]. This cell type-specific restriction later was attributed to hA3G expression levels, although hA3F also is a potent inhibitor of HIV-1 [16,17]. Interestingly, passage of Vif-minus HIV-1 in cells with hA3G expression does not lead to A3-resistant viruses [18]. Similarly, other retroviruses have been shown to encode inhibitors of A3 proteins [19,20,21], indicating that viral A3 inhibitors are essential for robust retroviral replication.

**Figure 1:**
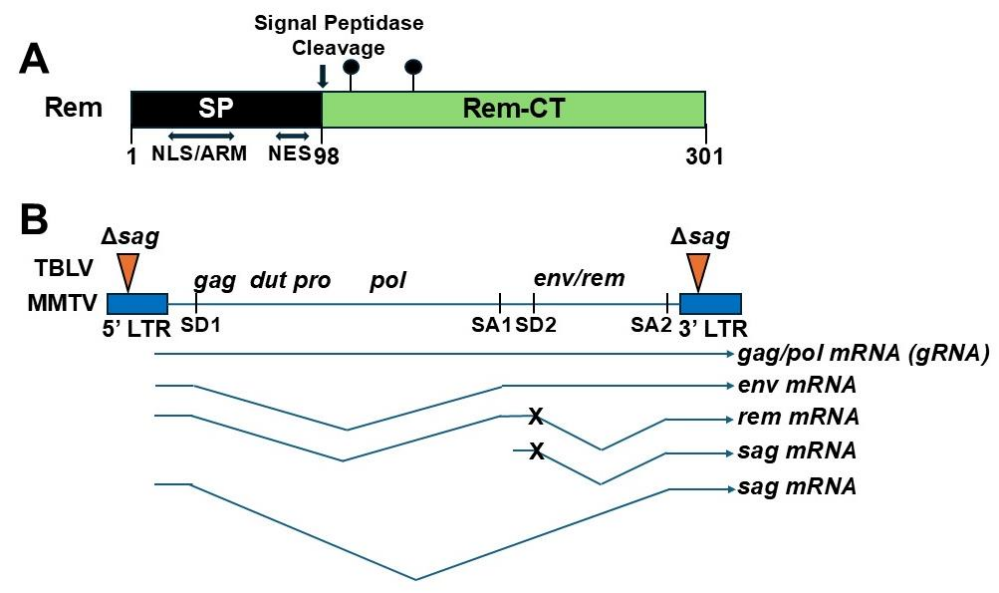
**Diagram of Rem and MMTV proviral structure.** (**A**) Rem structure. Rem is a 301-amino-acid protein that is synthesized at the ER membrane and then cleaved by signal peptidase as indicated by the arrow [27,29,33]. The 98-amino-acid signal peptide (SP; shown in black) has a nuclear localization signal (NLS) that also contains an arginine-rich motif (ARM). The ARM serves as an RNA-binding domain for the Rem-responsive element (RmRE) located at the 3′ end of all viral mRNAs [29]. The C-terminal cleavage product of Rem (Rem-CT; shown in green) is a deleted in-frame fusion product of the Env protein and retains two of the five glycosylation sites [27]. SP and uncleaved Rem are extracted from the ER membrane by retrotranslocation into the cytosol, after which both proteins can be imported into the nucleus through the NLS [27,33,34]. Rem-CT is localized primarily within the ER, but has an unusual trafficking pathway involving early and late endosomes without passing through the Golgi apparatus [28]. (**B**) Structure of the MMTV and TBLV proviruses and their mRNAs. The proviral long terminal repeats (LTRs) are shown in blue at the 5′ and 3′ ends of the provirus. Viral genes are in italics, and the two splice donor (SD1 and SD2) and splice acceptor (SA1 and SA2) sites are indicated. A variant of MMTV, TBLV, has a deletion within the LTRs that removes the C-terminal portion of the Sag-coding region (orange inverted triangles) and has a triplication that constitutes a T-cell-specific enhancer [35,36]. The genomic RNA (gRNA) is transcribed starting within the 5′ LTR and ends within the 3′ LTR. The gRNA also is the mRNA for the *gag*, *dut*, *pro*, and *po*l genes, which encode the non-glycosylated virion (Gag) proteins, the dUTPase, protease, integrase, and reverse transcriptase [37]. The V-shaped regions indicate introns within the spliced *env*, *rem*, and *sag* mRNAs. The superantigen (Sag) proteins are specified by two different singly spliced mRNAs, one originating in the 5′ LTR promoter and the other within the intragenic *env* promoter [38]. The X characters indicate the elimination of the SD2 site within the mutant MMTV and TBLV proviruses, which prevents the expression of Rem as well as Sag from the intragenic promoter [39].

Unlike HIV-1, MMTV requires replication in both B and T cells for transmission from maternal milk in the gut to its ultimate target tissue, the mammary gland [22,23,24]. Relative to human viruses, advantages of studying MMTV and its interactions with APOBECs include the ability to infect the native host with an intact immune system. Our experiments to understand MMTV pathogenesis led to the discovery of the multifunctional protein, regulator of export and expression of MMTV mRNA (Rem) [25,26]. Rem is a 301-amino-acid protein produced in the same reading frame as the viral envelope (Env) protein from a doubly spliced mRNA [25] (Figure 1). After synthesis in association with the endoplasmic reticulum (ER), Rem goes through an extraordinary journey through different parts of the cell [27,28]. The 98-amino-acid signal peptide (SP) is cleaved by signal peptidase and extracted from the ER membrane by retrotranslocation (Figure 1A). SP, which is shared between Env and Rem, is imported into the nucleus using its nuclear localization sequence (NLS), where it binds to the Rem-responsive element (RmRE) on all viral mRNAs to allow export from the nucleus (Figure 1B) [29]. SP (translated primarily from the more abundant singly spliced *env* mRNA) is an adapter for CRM1-mediated export of MMTV transcripts from the nucleus [25]. Although Rem is required for nuclear export of unspliced genomic MMTV RNA, a reporter vector including the 3′ end of the MMTV provirus was dependent on the RmRE and presence of Rem, but did not measure nuclear export of vector RNA [29,30]. Thus, the N-terminal part of Rem is an HIV-1 Rev-like adapter that likely has a role in both export and translation [25,30].

The function of the C-terminal cleavage product of Rem (Rem-CT or CT in standard retroviral nomenclature) has been more enigmatic. The requirement for Rem-CT activity was explored using engineered infectious MMTV clones with a mutation in the second splice donor site (MMTV-SD) to eliminate generation of the doubly spliced *rem* mRNA [31]. This strategy allowed SP production from *env* mRNA, but prevented Rem and Rem-CT expression (Figure 1B) [27]. SP is required for replication [25]. Infections with Rem-minus MMTV in tissue culture allowed virus production [32], indicating that Rem and its cleavage product are accessory proteins.

Infection of BALB/c mice with wild-type MMTV or the Rem-minus mutant allowed mammary tumor production, but with a longer latency and lower incidence in mutant-injected animals [31]. Sequence analysis of tumor-derived proviruses revealed that proviruses lacking Rem expression had a statistically higher number of G-to-A mutations on the plus strand compared to wild-type MMTV proviruses. Furthermore, the increased mutations were primarily within the TYC motif typical of mA3 [31]. A number of proviruses lost the SD mutation due to recombination with endogenous MMTVs (*Mtv*s), which are often expressed in lymphocytes [40,41]. MMTV recombinants had significantly higher mutations in the WRC context attributed to mAID [31], suggesting that restoration of Rem expression was needed to reconstitute an infectious virus after APOBEC mutagenesis. These results also are consistent with mAID and mA3 expression in lymphocytes [42,43,44], which are required for MMTV replication and transmission [23,45]. Since SP is synthesized from *env* mRNA [25,27], these data suggested that Rem C-terminal sequences block the mutagenic effects of multiple APOBECs [31].

## 4. Type B Leukemogenic Virus and Rem Antagonism of APOBECs

MMTV is a complex retrovirus that encodes multiple genes at the 3′ end of the genome, including *env*, *rem*, and *sag* (Figure 1B) [25]. Because *env* and *rem* genes are in the same reading frame, *rem* mutations potentially will affect MMTV replication due to disruption of Env function. The site mutated in the 3′ MMTV splice donor site will not affect Env production, but is needed for Sag expression from the *env* intragenic promoter [38] as well as Rem or Rem-CT production [25] (Figure 1B). Sag is required for lymphocyte-mediated MMTV transmission to mammary glands in vivo [22,23,24]. Therefore, it is possible that loss of Sag, rather than Rem, contributes to the mutagenic phenotype observed in the MMTV splice donor mutant.

To distinguish the effects of Rem and Sag on proviral mutagenesis, an MMTV variant, type B leukemogenic virus (TBLV), was used to generate infectious proviral clones with the splice donor mutation. Unlike MMTV, TBLV does not encode functional Sag for replication or induction of tumors [35,39]. The TBLV *sag* gene is inactivated by an LTR deletion as well as triplication of flanking sequences to constitute a T-cell enhancer [39]. Moreover, TBLV does not require mature B-2 cells for tumor induction, as demonstrated by infection of mice lacking µ immunoglobulin heavy chain production [46], whereas MMTV does [45]. These experiments indicated that studies with Sag-independent TBLV could distinguish between the effects of Sag and Rem.

Wild-type TBLV and mutant TBLV lacking Rem expression were used for infection of BALB/c mice and induction of tumors. Unlike the results obtained with Sag-dependent MMTV, no difference was observed in the latency or incidence of T-cell lymphomas induced by wild-type or Rem-mutant TBLV. However, proviral loads were reduced in the absence of Rem expression [31]. High-throughput sequencing of tumor-derived viral DNA revealed a dramatic increase in G-to-A mutations on the viral DNA plus strand, particularly in proviruses that restored the splice site specific to Rem production by recombination with endogenous *Mtv*s [31]. Consistent with sequencing results from Rem-mutant MMTV proviruses, mutations occurred primarily in the TYC and WRC motifs preferred by mA3 and mAID, respectively. However, the numbers of mutations were higher in the TYC compared to the WRC motif in the absence of Rem. The appearance of revertants after infections with either Rem-mutant MMTV or TBLV revealed the importance of specific spliced mRNAs and their translation products for successful replication in vivo. The numbers of mutations were highly proportional to the numbers of recombinants isolated from tumors [31], suggesting that recombination of exogenous MMTVs with endogenous *Mtv*s was a mechanism to recover from the effects of APOBEC-mediated damage. Because TBLV does not make functional Sag protein [39], these experiments predicted that Rem, rather than Sag, limits APOBEC-mediated mutagenesis in vivo [31].

## 5. Genetic Evidence for Rem-Mediated AID Antagonism

Previous experiments have shown that mA3 is an inhibitor of MMTV replication in C57BL/6 (B6) mice [47,48]. However, current data suggest that MMTV infection of B6 mice does not result in proviral mutations regardless of Rem expression [46] (see below). Because MMTV replicates in both B and T cells for trafficking to the mammary gland [22], BALB/c mice with a knockout mutation in the *Aicda* (AID) gene were derived and infected with wild and Rem-defective MMTV independently. Mouse AID expressed in B-2 lymphocytes is known to be involved in immunoglobulin gene hypermutation and class switch recombination [43]. MMTV has a low replication profile in lymphocytes, does not cause viremia, and is very difficult to detect prior to replication in lactating mammary glands and tumors [37,49]. In contrast, cells from mammary tumors are uniformly infected [50,51]. *Aicda^−/−^* tumors induced by Rem-defective MMTV had longer latency and a lower incidence than tumors induced by wild-type MMTV. Unlike results obtained in wild-type BALB/c mice, there was no difference in proviral load in tumors from mAID-knockout animals after infection with wild-type or Rem-defective virus [31]. These results suggested that the absence of Rem compromised tumorigenesis in mAID-knockout mice without affecting proviral copy numbers. MMTV-induced tumors are known to be affected by mutations within the viral enhancer [35,52,53,54], which is required to activate specific oncogenes, such as *Wnt1*, *Fgf3*, *and c-Myc* [51,55,56]. One possibility is that Rem expression affects selection for viral enhancer mutations.

To determine the effect of mAID deficiency on Rem-associated mutations, tumor DNA was used for PCR of the envelope region at the 3′ end of MMTV proviruses. Products from MMTV-induced *Aicda^−/−^* tumors were cloned, sequenced, and compared to samples obtained from infected wild-type BALB/c mice. As anticipated, no differences were observed between proviral mutations within the WRC motifs in either wild-type or recombinant proviruses isolated from mAID-knockout tumors [31]. Unexpectedly, the absence of mAID expression also eliminated the increased proviral mutations in the TYC motif typical of mA3 in MMTV mutant (Rem-null) infections of wild-type BALB/c mice. These results were consistent with the idea that mAID is required for both WRC and TYC-motif mutations. In addition, mutations in the SYC motif were elevated in MMTV recombinants with endogenous *Mtv*s compared to either wild-type MMTV or MMTV-SD non-recombinant proviruses. Although SYC motifs have been considered low-level targets of mAID or hAID on the immunoglobulin locus [57,58], the discrepancy between these results and those obtained for the WRC motifs suggests that SYC is the target of another deaminase on the MMTV genome. Alternatively, mA3 target specificity may be changed by protein adapters and/or post-translational modifications.

## 6. Rem and Endogenous Mtvs

Endogenous *Mtv*s presumably were acquired by infections of germline cells with exogenous MMTVs [59]. Most *Mtv*s, distinguished by their independent insertion sites with separate numbers [60], have *env* gene mutations that would prevent infectious virion production and fusion functions [61]. Such *Mtv*s have lost Env function by deletion or point mutation [61,62], which potentially affects Rem activity due to *rem* being an in-frame deletion of *env* [25,26]. Some *Mtv env* deletions exactly coincide with one of the *rem* gene introns [61] (see Figure 1B), indicating incorporation into an exogenous MMTV by recombination between packaged viral genomic and *rem* mRNA prior to endogenization [61]. G-to-A mutations typical of APOBECs that result in stop codons are present in *env*, but not *rem*, in most *Mtv* proviruses [61]. These results suggest that endogenous *Mtv*s have been selected for their retention of Rem expression in mice. One idea is that APOBEC regulation by Rem has affected *Mtv* retention similar to the effects of *Mtv* Sag expression in shaping the T-cell repertoire of the mouse [23,63].

## 7. Mechanism of Rem-Mediated APOBEC Antagonism

HIV-1 Vif inhibits hA3G and hA3F activity by acting as an adapter to a ubiquitin E3 ligase complex [64,65]. Proteasomal degradation of the A3 proteins prevented their incorporation into viral particles. Without virion incorporation, the A3 enzymes could not inhibit reverse transcription or cause deamination of reverse transcripts. To determine if MMTV Rem used a similar mechanism, Rem expression constructs were transfected together with constructs for mA3 or mAID. Surprisingly, mAID was degraded in a dose-dependent manner, whereas mA3 was not. Proteasomal degradation of AID in the presence of Rem was confirmed by rescue of AID levels after incubation of transfected cells in the presence of the proteasomal inhibitor MG132 [31]. Furthermore, co-transfection of TBLV-WT proviruses expressing Rem in human Jurkat cells with tagged murine AID showed degradation of AID. Co-transfection of TBLV mutants lacking Rem expression with mAID revealed no evidence of reduced AID levels. Degradation was proportional to Rem expression [31], consistent with the idea that Rem reduces mAID, but not mA3 levels.

Multiple investigators have shown that mA3 is incorporated into wild-type MMTV [31,47,48] and MLV [66,67,68] virions, indicating that viral antagonists do not prevent mA3 packaging. In contrast, mAID is not packaged into Abelson MLV [69], and experiments to detect AID in MMTV virions have not been successful [31]. One idea is that mAID-mediated viral mutagenesis occurs outside of virions. Alternatively, AID may influence the activity of packaged mA3 or a cofactor that determines viral target specificity.

## 8. Mouse Strain-Specific Differences in APOBEC-Mediated Proviral Mutagenesis

Previous studies have reported that proviral mutations in the mA3 motif are not observed after MMTV infections of B6 mice [47]. Nevertheless, these experiments were performed with MMTV (RIII strain) that retained Rem expression, which would inhibit APOBEC-mediated mutations [31]. To avoid the potential effects of the mutation on both Rem and Sag expression that complicate results with MMTV, wild-type and Rem mutant TBLV were used for infection of wild-type B6 mice. Infections with Rem-mutant TBLV accelerated the appearance of T-cell lymphomas relative to TBLV-WT infections in both wild-type and AID-knockout mice. The incidences of tumors induced by the wild type and the Rem mutant were 20–30% and 80–90%, respectively, and the latency of lymphomas was delayed in tumors induced by wild-type TBLV [31,46]. Since TBLV does not make Sag [35,39], these results imply that loss of Rem expression provides an advantage for viral tumorigenesis in B6 mice.

One possibility is that Rem-mutant proviruses have increased mutations induced by APOBECs that cripple virus replication or increase the activity of LTR enhancers on nearby oncogenes. To test this, the 3′ end of proviruses from wild-type and *Aicda*^−/−^ tumors induced by wild-type and Rem-mutant TBLV in B6 mice was subjected to PCR, cloning, and sequencing. In contrast to the results obtained for TBLV-infected BALB/c mice [31], analysis of the mutations/clone showed no differences between wild-type and Rem-mutant proviruses derived from wild-type or AID-knockout B6 mice for the WRC and TYC motifs associated with AID and mA3, respectively [46]. TYC mutations typical of mA3 were the most abundant sequence changes observed in any of the conditions tested (Table 1).

In a subsequent experiment, wild-type, *Aicda*^−/−^, and *mA3/Aicda*^−/−^ (double-knockout) mice were used for infection by clonal TBLV proviruses with or without Rem expression. Tumor DNA was used for PCR to encompass the *env*/3′ LTR region and subjected to high-throughput sequencing. As previously observed, G-to-A changes on the viral plus strand were the most abundant [46]. Analysis of the results indicated that no statistical differences were observed between levels of G-to-A changes when comparing mutations in wild-type and Rem-minus proviruses in either wild-type B6 or AID-knockout mice. The G-to-A changes were significantly reduced in mA3/AID-double knockout tumors compared to those obtained from wild-type B6 or AID single-knockout animals. These experiments suggest that mA3 is a TBLV mutagen in B6 mice. However, significant numbers of G-to-A mutations were detected in the absence of both mAID and mA3 expression, consistent with additional deaminase activity on TBLV proviruses [31].

## 9. Apobec and Rem Expression in Mice

How can the differences in APOBEC mutagenesis of proviruses in BALB/c and B6 mice be explained? These mouse strains have multiple differences in their immune systems since BALB/c mice are known to have Th2-biased immunity, whereas B6 mice have Th1-biased cell-mediated responses [70,71]. These strains also have differences in mA3 and AID expression [46]. BALB/c mice express two isoforms of *mA3* mRNA, one that is full-length and one that is missing exon5 (Δexon5). In contrast, B6 mice express only the Δexon5 isoform [67,72,73]. Furthermore, the B6 gene has a xenotropic mouse leukemia virus (MLV) LTR in the vicinity of the *mA3* gene. This LTR is missing from the BALB/c *mA3* gene, suggesting that the lower levels of *mA3* transcripts in this strain are due to lack of the MLV LTR enhancer [74,75,76,77].

In addition to the higher *mA3* mRNA levels in B6 mice, there are 15 amino acid differences specified by the *mA3* gene of BALB/c mice. The majority of these amino acids (11 of 15) are under strong positive selection [9,73], consistent with their contribution to antiviral resistance. The mA3 protein has two deaminase domains: one in the N-terminus, which is catalytically active, and a second enzymatically inactive domain in the C-terminus [78]. The N-terminal mA3 domain mutations found in multiple mouse strains have been associated with substrate selection, rather than deaminase activity. This conclusion was based on comparisons to the domain structure and activities of the analogous hA3G amino acids [9]. The C-terminal mA3 mutations that differ between BALB/c and B6 also are under positive selection, but are not associated with the known function of this domain in virion incorporation [79,80]. The preferred target site for BALB/c mA3 is XTC, whereas the B6 enzyme preferentially mutates cytidines in the TYC motif [81]. Although experiments indicate that the high polymerization by MMTV reverse transcriptase interferes with mA3 activity [82], both endogenous *Mtv*s and exogenous MMTVs show evidence of proviral DNA hypermutation [9,31,46].

Published data indicate that mA3 is under positive selection [9,73], suggesting that this deaminase is responding to interactions with mouse pathogens. No information is available for AID. The levels of mAID protein are higher in BALB/c relative to B6 splenocytes and inducible within 48 h after induction by lipopolysaccharide (LPS) and IL-4 [46]. On the other hand, mA3 protein levels are ~5-fold higher than those in BALB/c in unstimulated splenocytes. B6 mA3 can be induced by LPS and IL-4 15-to−30-fold over BALB/c baseline expression, whereas BALB/c mA3 levels are elevated ~5-fold under the same conditions [46]. In summary, higher levels of AID relative to mA3 in lymphocytes correlate with responsiveness of TBLV proviral mutations to Rem expression (Table 1).

## 10. Model for Rem Antagonism of APOBECs

Since Rem affects mAID, but not mA3 levels, how does Rem affect MMTV proviral mutations that are typical of mA3? One possibility is that the AID-to-mA3 ratio contributes to the higher levels of mutations observed in TBLV proviruses after infections of BALB/c mice relative to those observed in B6 mice [46]. Moreover, Rem reduces AID levels by proteasomal degradation [31], leading to decreased AID/mA3 ratios in cells infected with wild-type MMTV or TBLV. Under this condition, few mutations are observed in BALB/c mice, which have higher basal levels of AID [46]. However, when Rem is absent (i.e., when the mAID/mA3 ratio is increased), infections of BALB/c mice with splice-donor mutant viruses show increased proviral G-to-A changes [31]. In B6 mice, which have lower baseline levels of mAID relative to mA3, little difference is observed in the presence and absence of Rem expression (Table 1) [46]. Our hypothesis is that the relative levels of AID are too low in some B6 cell types to affect mA3 function.

Genetic data also suggest that AID controls mA3 activity. MMTV infection of BALB/c AID-knockout mice with viruses with or without Rem-coding potential revealed that tumor-associated proviruses showed no significant mutational differences [31]. One interpretation of the loss of mA3-like proviral mutations in the absence of mAID expression is that there is an mA3/AID interaction. Published data indicate that hA3 enzymes are present in cytosolic bodies, such as stress granules and P-bodies [83,84], which are likely sites for interactions with hAID. Therefore, our untested hypothesis is that Rem-mediated degradation of AID will alter the localization and association between mA3 and mAID. This interaction (either direct or indirect through adapters) would then control the mutagenic activity of both enzymes.

What are the cytosolic bodies containing both deaminases? Previous reports have indicated that hA3G is associated with P-bodies and stress granules [84,85], which are dynamic structures enriched for RNAs, protein chaperones, RNA-binding proteins, and translation factors [86,87]. Rem, mA3, and mAID are all RNA-binding proteins [29,88]. Furthermore, hA3B has been shown to interact with polyA-binding protein C1 (PABPC1) and induce signaling through protein kinase RNA-activated (PKR) (also known as eukaryotic translation initiation factor 2-alpha kinase 2 or EIF2AK2) [83]. This signal occurs after various kinds of stress, including viral infection, ER stress, and the unfolded protein response [89,90,91], which leads to translational shutoff [83,92]. In addition, A3B shields viral mRNA from RNase L cleavage by assisting protein–RNA condensation using the stress granule assembly factor G3BP1 [83]. Cytoplasmic AID has been observed to interact with translation factors, such as eEF1A [93], and is tethered outside the nucleus by the chaperone protein Hsp90 [94,95]. Hsp90 has been associated with stress granule disassembly [96]. Furthermore, RNA sensors involved in innate immunity, such as melanoma differentiation associated gene-5 (MDA-5) and oligoadenylate synthetases (OASs), are localized to stress granules and other cytoplasmic ribonucleoprotein condensates [97,98]. Experimental results using multiple different viruses have shown the concentration of foreign nucleic acids in various RNA–DNA–antiviral protein condensates to modulate cell signaling and pathogen replication (reviewed in [99,100]). Therefore, this evidence is consistent with the A3 association with cytosolic bodies and innate immune responses.

Severe acute respiratory syndrome coronavirus-2 (SARS-CoV-2) has been reported to sustain multiple mutations induced by APOBECs [101,102,103,104]. Recent data indicate that SARS-CoV-2 nucleocapsid (N) protein acts as an adapter to both adenosine deaminase acting on RNA (ADAR) and APOBECs. These interactions localize the N packaging protein–deaminase complexes to stress granules to increase—not decrease—mutagenesis of viral RNA, presumably to provide viral diversity [105]. HTLV-1 nucleocapsid protein (NC) also appears to inhibit viral genome packaging of A3 proteins [106], yet has a low impact on HTLV-1 mutagenesis [107]. These observations suggest that APOBEC as well as ADAR enzymes colocalize with cytoplasmic complexes associated with stress, allowing them access to viral RNAs outside of virion particles. APOBEC interactions with viral proteins may lead to increased or decreased mutations. It remains unclear whether SARS-CoV-2 and HTLV-1 nucleocapsid interactions with APOBECs are mechanistically distinct.

**Figure 2:**
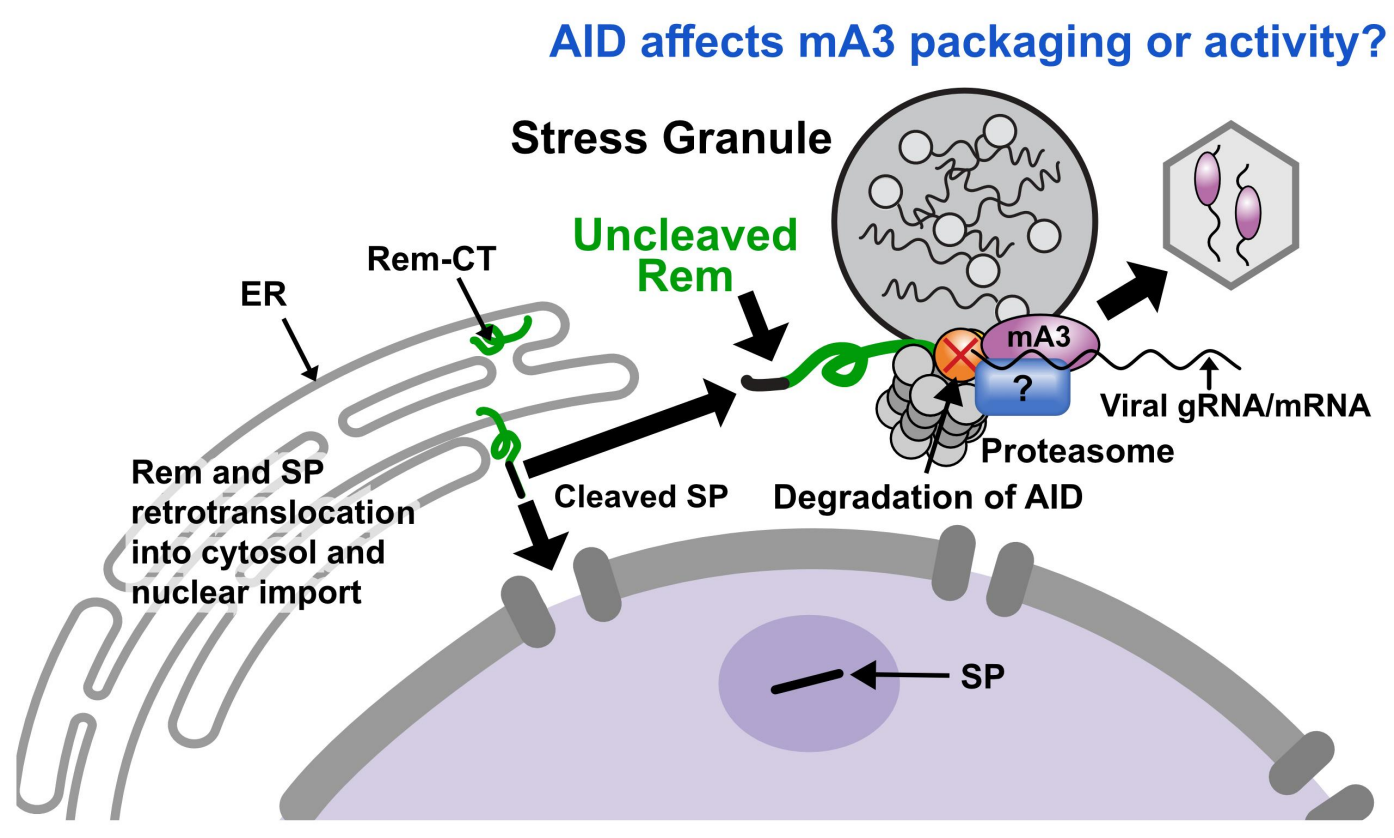
**Model for Rem interactions with AID and mouse APOBEC3.** Uncleaved Rem is synthesized at the ER membrane and extracted by retrotranslocation prior to cleavage into SP and Rem-CT [27]. The cleavage products SP and Rem-CT traffic to the nucleus and ER, respectively [25,27]. SP and Rem contain an NES that allows nuclear shuttling [25,26]. Rem serves as an adapter between the proteasome (gray double hexamer) and AID (orange) [31]. AID is polyubiquitylated by cellular enzymes, and the red X indicates AID degradation. Rem likely is associated with viral mRNAs exported from the nucleus through binding to the Rem-responsive element [29]. AID and mA3 (purple) are expected to interact with viral genomic RNA (gRNA) and mRNA as well as each other in association with cytosolic bodies, e.g., stress granules. It is unclear whether additional proteins (blue rectangle with question mark) mediate the interaction between AID and mA3 or act as cofactors for enzymatic activity. Retention of viral mRNA in cytosolic bodies by AID and mA3 may decrease virion packaging (gray hexagon) or mRNA association with ribosomes.

Does the interaction of mA3 and mAID affect MMTV mutagenesis and inhibition of replication? If so, how? One model consistent with current data is that cellular stress, including viral infection, leads to sequestration of viral mRNAs and cellular deaminases in cytosolic stress granules or a related condensate (Figure 2). If A3 enzymes shield viral RNAs from RNase L degradation as proposed [83], the A3/AID deaminases would have an opportunity to associate with genomic RNA prior to packaging. Interactions of A3 and AID may influence the activity of other deaminases within cytoplasmic condensates and/or prolong viral RNA sequestration to prevent their translation. Moreover, mRNA translation of host immune genes may be affected since APOBECs have the ability to mutate both host and viral RNAs [101,108,109,110]. Multiple aspects of these ideas are untested. Regardless of the details, this model provides a framework for understanding the multiple mechanisms that cytidine deaminases use for viral inhibition, while promoting the emergence of variants [3,5,14]. Studies of viral antagonists, such as MMTV Rem, offer a window into these mechanisms.

## Figures and Tables

**Table 1:** TBLV proviral mutations in response to Rem expression in BALB/c and B6 mice.

Mouse Strain	Relative AID Splenocyte Levels	Relative mA3 Splenocyte Levels	Dominant TBLV APOBEC Mutations	Effect of Rem Expression on APOBEC Mutations
BALB/c	High	Low	TYC	Reduced
B6	Low	High	TYC	No effect

## Data Availability

No new data were created or analyzed in this study.

## Abbreviations

The following abbreviations are used in this manuscript:

A3	APOBEC3
ADAR	Adenosine deaminase acting on RNA
*Aicda*	Gene encoding AID
AID	Activation-induced cytidine deaminase
APOBEC	Apolipoprotein B mRNA-editing catalytic polypeptide-like
CA	Capsid
CRM-1	Chromosome maintenance 1, also known as Exportin 1
eEF1A	Eukaryotic elongation factor 1A
Env	Envelope
ER	Endoplasmic reticulum
HIV-1	Human immunodeficiency virus type 1
HTLV-1	Human T-cell leukemia virus type 1
IL-4	Interleukin-4
LPS	Lipopolysaccharide
LTR	Long terminal repeat
MLV	Mouse leukemia virus
MMTV	Mouse mammary tumor virus
*Mtv*	Endogenous mouse mammary tumor virus
NC	Nucleocapsid
NLS	Nuclear localization sequence
PABPC1	PolyA-binding protein C1
PKR	Protein kinase RNA-activated
RmRE	Rem-responsive element
SARS-CoV-2	Severe acute respiratory syndrome coronavirus 2
SD	Splice donor
SP	MMTV-encoded signal peptide
TBLV	Type B leukemogenic virus

## References

[1] Beirag N., Varghese P.M., Kishore U. (2025). Innate Immune Response to Viral Infection. Adv. Exp. Med. Biol..

[2] Lam N., Lee Y., Farber D.L. (2024). A Guide to Adaptive Immune Memory. Nat. Rev. Immunol..

[3] Harris R.S., Dudley J.P. (2015). APOBECs and Virus Restriction. Virology.

[4] Knisbacher B.A., Gerber D., Levanon E.Y. (2016). DNA Editing by APOBECs: A Genomic Preserver and Transformer. Trends Genet..

[5] Xu W.K., Byun H., Dudley J.P. (2020). The Role of APOBECs in Viral Replication. Microorganisms.

[6] Harari A., Ooms M., Mulder L.C.F., Simon V. (2009). Polymorphisms and Splice Variants Influence the Antiretroviral Activity of Human APOBEC3H. J. Virol..

[7] Anant S., MacGinnitie A.J., Davidson N.O. (1995). Apobec-1, the Catalytic Subunit of the Mammalian Apolipoprotein B mRNA Editing Enzyme, Is a Novel RNA-Binding Protein. J. Biol. Chem..

[8] Jern P., Stoye J.P., Coffin J.M. (2007). Role of APOBEC3 in Genetic Diversity among Endogenous Murine Leukemia Viruses. PLoS Genet..

[9] Shaffer E., Boso G., Sadjadpour R., Yedavalli V.V.S.R.K., Lam O., Kozak C.A. (2025). Apobec3 Shows Rapid Evolution in House Mouse Subspecies and Unusual Hypermutation Patterns of Endogenous Mouse Mammary Tumor Viruses. J. Virol..

[10] Lecossier D., Bouchonnet F., Clavel F., Hance A.J. (2003). Hypermutation of HIV-1 DNA in the Absence of the Vif Protein. Science.

[11] Sheehy A.M., Gaddis N.C., Choi J.D., Malim M.H. (2002). Isolation of a Human Gene That Inhibits HIV-1 Infection and Is Suppressed by the Viral Vif Protein. Nature.

[12] Guo F., Cen S., Niu M., Saadatmand J., Kleiman L. (2006). Inhibition of tRNA_3_(Lys)-Primed Reverse Transcription by Human APOBEC3G during Human Immunodeficiency Virus Type 1 Replication. J. Virol..

[13] Schröfelbauer B., Yu Q., Zeitlin S.G., Landau N.R. (2005). Human Immunodeficiency Virus Type 1 Vpr Induces the Degradation of the UNG and SMUG Uracil-DNA Glycosylases. J. Virol..

[14] Dudley J.P. (2023). APOBECs: Our Fickle Friends?. PLoS Pathog.

[15] Sova P., Volsky D.J. (1993). Efficiency of Viral DNA Synthesis during Infection of Permissive and Nonpermissive Cells with Vif-Negative Human Immunodeficiency Virus Type 1. J. Virol..

[16] Liddament M.T., Brown W.L., Schumacher A.J., Harris R.S. (2004). APOBEC3F Properties and Hypermutation Preferences Indicate Activity against HIV-1 in Vivo. Curr. Biol..

[17] Wiegand H.L., Doehle B.P., Bogerd H.P., Cullen B.R. (2004). A Second Human Antiretroviral Factor, APOBEC3F, Is Suppressed by the HIV-1 and HIV-2 Vif Proteins. EMBO J..

[18] Miyagi E., Kao S., Fumitaka M., Buckler-White A., Plishka R., Strebel K. (2017). Long-Term Passage of Vif-Null HIV-1 in CD4+ T Cells Expressing Sub-Lethal Levels of APOBEC Proteins Fails to Develop APOBEC Resistance. Virology.

[19] Jaguva Vasudevan A.A., Perkovic M., Bulliard Y., Cichutek K., Trono D., Häussinger D., Münk C. (2013). Prototype Foamy Virus Bet Impairs the Dimerization and Cytosolic Solubility of Human APOBEC3G. J. Virol..

[20] Yoshikawa R., Takeuchi J.S., Yamada E., Nakano Y., Misawa N., Kimura Y., Ren F., Miyazawa T., Koyanagi Y., Sato K. (2017). Feline Immunodeficiency Virus Evolutionarily Acquires Two Proteins, Vif and Protease, Capable of Antagonizing Feline APOBEC3. J. Virol..

[21] Stavrou S., Nitta T., Kotla S., Ha D., Nagashima K., Rein A.R., Fan H., Ross S.R. (2013). Murine Leukemia Virus Glycosylated Gag Blocks Apolipoprotein B Editing Complex 3 and Cytosolic Sensor Access to the Reverse Transcription Complex. Proc. Natl. Acad. Sci. USA.

[22] Golovkina T.V., Dudley J.P., Ross S.R. (1998). B and T Cells Are Required for Mouse Mammary Tumor Virus Spread within the Mammary Gland. J. Immunol..

[23] Golovkina T.V., Chervonsky A., Dudley J.P., Ross S.R. (1992). Transgenic Mouse Mammary Tumor Virus Superantigen Expression Prevents Viral Infection. Cell.

[24] Held W., Shakhov A.N., Izui S., Waanders G.A., Scarpellino L., MacDonald H.R., Acha-Orbea H. (1993). Superantigen-Reactive CD4+ T Cells Are Required to Stimulate B Cells after Infection with Mouse Mammary Tumor Virus. J. Exp. Med..

[25] Mertz J.A., Simper M.S., Lozano M.M., Payne S.M., Dudley J.P. (2005). Mouse Mammary Tumor Virus Encodes a Self-Regulatory RNA Export Protein and Is a Complex Retrovirus. J. Virol..

[26] Indik S., Günzburg W.H., Salmons B., Rouault F. (2005). A Novel, Mouse Mammary Tumor Virus Encoded Protein with Rev-like Properties. Virology.

[27] Byun H., Halani N., Mertz J.A., Ali A.F., Lozano M.M., Dudley J.P. (2010). Retroviral Rem Protein Requires Processing by Signal Peptidase and Retrotranslocation for Nuclear Function. Proc. Natl. Acad. Sci. USA.

[28] Xu W.K., Gou Y., Lozano M.M., Dudley J.P. (2021). Unconventional P97/VCP-Mediated Endoplasmic Reticulum-to-Endosome Trafficking of a Retroviral Protein. J. Virol..

[29] Mertz J.A., Chadee A.B., Byun H., Russell R., Dudley J.P. (2009). Mapping of the Functional Boundaries and Secondary Structure of the Mouse Mammary Tumor Virus Rem-Responsive Element. J. Biol. Chem..

[30] Mertz J.A., Lozano M.M., Dudley J.P. (2009). Rev and Rex Proteins of Human Complex Retroviruses Function with the MMTV Rem-Responsive Element. Retrovirology.

[31] Singh G.B., Byun H., Ali A.F., Medina F., Wylie D., Shivram H., Nash A.K., Lozano M.M., Dudley J.P. (2019). A Protein Antagonist of Activation-Induced Cytidine Deaminase Encoded by a Complex Mouse Retrovirus. mBio.

[32] Mustafa F., Lozano M., Dudley J.P. (2000). C3H Mouse Mammary Tumor Virus Superantigen Function Requires a Splice Donor Site in the Envelope Gene. J. Virol..

[33] Dultz E., Hildenbeutel M., Martoglio B., Hochman J., Dobberstein B., Kapp K. (2008). The Signal Peptide of the Mouse Mammary Tumor Virus Rem Protein Is Released from the Endoplasmic Reticulum Membrane and Accumulates in Nucleoli. J. Biol. Chem..

[34] Das P., Xu W.K., Gautam A.K.S., Lozano M.M., Dudley J.P. (2022). A Retrotranslocation Assay That Predicts Defective VCP/P97-Mediated Trafficking of a Retroviral Signal Peptide. mBio.

[35] Ball J.K., Diggelmann H., Dekaban G.A., Grossi G.F., Semmler R., Waight P.A., Fletcher R.F. (1988). Alterations in the U3 Region of the Long Terminal Repeat of an Infectious Thymotropic Type B Retrovirus. J. Virol..

[36] Mertz J.A., Mustafa F., Meyers S., Dudley J.P. (2001). Type B Leukemogenic Virus Has a T-Cell-Specific Enhancer That Binds AML-1. J. Virol..

[37] Dudley J.P., Golovkina T.V., Ross S.R. (2016). Lessons Learned from Mouse Mammary Tumor Virus in Animal Models. ILAR J..

[38] Miller C.L., Garner R., Paetkau V. (1992). An Activation-Dependent, T-Lymphocyte-Specific Transcriptional Activator in the Mouse Mammary Tumor Virus Env Gene. Mol. Cell. Biol..

[39] Mustafa F., Bhadra S., Johnston D., Lozano M., Dudley J.P. (2003). The Type B Leukemogenic Virus Truncated Superantigen Is Dispensable for T-Cell Lymphomagenesis. J. Virol..

[40] Henrard D., Ross S.R. (1988). Endogenous Mouse Mammary Tumor Virus Is Expressed in Several Organs in Addition to the Lactating Mammary Gland. J. Virol..

[41] Barnett A., Mustafa F., Wrona T.J., Lozano M., Dudley J.P. (1999). Expression of Mouse Mammary Tumor Virus Superantigen mRNA in the Thymus Correlates with Kinetics of Self-Reactive T-Cell Loss. J. Virol..

[42] Ito S., Nagaoka H., Shinkura R., Begum N., Muramatsu M., Nakata M., Honjo T. (2004). Activation-Induced Cytidine Deaminase Shuttles between Nucleus and Cytoplasm like Apolipoprotein B mRNA Editing Catalytic Polypeptide 1. Proc. Natl. Acad. Sci. USA.

[43] Muramatsu M., Kinoshita K., Fagarasan S., Yamada S., Shinkai Y., Honjo T. (2000). Class Switch Recombination and Hypermutation Require Activation-Induced Cytidine Deaminase (AID), a Potential RNA Editing Enzyme. Cell.

[44] Tsukimoto S., Hakata Y., Tsuji-Kawahara S., Enya T., Tsukamoto T., Mizuno S., Takahashi S., Nakao S., Miyazawa M. (2022). Distinctive High Expression of Antiretroviral APOBEC3 Protein in Mouse Germinal Center B Cells. Viruses.

[45] Beutner U., Kraus E., Kitamura D., Rajewsky K., Huber B.T. (1994). B Cells Are Essential for Murine Mammary Tumor Virus Transmission, but Not for Presentation of Endogenous Superantigens. J. Exp. Med..

[46] Byun H., Singh G.B., Xu W.K., Das P., Reyes A., Battenhouse A., Wylie D.C., Santiago M.L., Lozano M.M., Dudley J.P. (2024). Apobec-Mediated Retroviral Hypermutation in Vivo Is Dependent on Mouse Strain. PLoS Pathog..

[47] Okeoma C.M., Lovsin N., Peterlin B.M., Ross S.R. (2007). APOBEC3 Inhibits Mouse Mammary Tumour Virus Replication in Vivo. Nature.

[48] Okeoma C.M., Huegel A.L., Lingappa J., Feldman M.D., Ross S.R. (2010). APOBEC3 Proteins Expressed in Mammary Epithelial Cells Are Packaged into Retroviruses and Can Restrict Transmission of Milk-Borne Virions. Cell Host Microbe.

[49] Bhadra S., Lozano M.M., Payne S.M., Dudley J.P. (2006). Endogenous MMTV Proviruses Induce Susceptibility to Both Viral and Bacterial Pathogens. PLoS Pathog..

[50] Nusse R., van Ooyen A., Cox D., Fung Y.K., Varmus H. (1984). Mode of Proviral Activation of a Putative Mammary Oncogene (Int-1) on Mouse Chromosome 15. Nature.

[51] Peters G., Brookes S., Smith R., Dickson C. (1983). Tumorigenesis by Mouse Mammary Tumor Virus: Evidence for a Common Region for Provirus Integration in Mammary Tumors. Cell.

[52] Michalides R., Wagenaar E., Weijers P. (1985). Rearrangements in the Long Terminal Repeat of Extra Mouse Mammary Tumor Proviruses in T-Cell Leukemias of Mouse Strain GR Result in a Novel Enhancer-like Structure. Mol. Cell. Biol..

[53] Hsu C.L., Fabritius C., Dudley J. (1988). Mouse Mammary Tumor Virus Proviruses in T-Cell Lymphomas Lack a Negative Regulatory Element in the Long Terminal Repeat. J. Virol..

[54] Bhadra S., Lozano M.M., Dudley J.P. (2005). Conversion of Mouse Mammary Tumor Virus to a Lymphomagenic Virus. J. Virol..

[55] Nusse R., Varmus H.E. (1982). Many Tumors Induced by the Mouse Mammary Tumor Virus Contain a Provirus Integrated in the Same Region of the Host Genome. Cell.

[56] Rajan L., Broussard D., Lozano M., Lee C.G., Kozak C.A., Dudley J.P. (2000). The C-Myc Locus Is a Common Integration Site in Type B Retrovirus-Induced T-Cell Lymphomas. J. Virol..

[57] Wei L., Chahwan R., Wang S., Wang X., Pham P.T., Goodman M.F., Bergman A., Scharff M.D., MacCarthy T. (2015). Overlapping Hotspots in CDRs Are Critical Sites for V Region Diversification. Proc. Natl. Acad. Sci. USA.

[58] MacCarthy T., Kalis S.L., Roa S., Pham P., Goodman M.F., Scharff M.D., Bergman A. (2009). V-Region Mutation in Vitro, in Vivo, and in Silico Reveal the Importance of the Enzymatic Properties of AID and the Sequence Environment. Proc. Natl. Acad. Sci. USA.

[59] Dopkins N., Nixon D.F. (2024). Activation of Human Endogenous Retroviruses and Its Physiological Consequences. Nat. Rev. Mol. Cell Biol..

[60] Kozak C., Peters G., Pauley R., Morris V., Michalides R., Dudley J., Green M., Davisson M., Prakash O., Vaidya A. (1987). A Standardized Nomenclature for Endogenous Mouse Mammary Tumor Viruses. J. Virol..

[61] Lam O., Shaffer E., Boso G., Kozak C.A. (2025). Intact, Recombinant, and Spliced Forms of Endogenous Mouse Mammary Tumor Viruses in Inbred and Wild Mice. J. Virol..

[62] Salmons B., Knedlitschek G., Kennedy N., Groner B., Ponta H. (1986). The Endogenous Mouse Mammary Tumour Virus Locus Mtv-8 Contains a Defective Envelope Gene. Virus Res..

[63] Held W., Waanders G.A., MacDonald H.R., Acha-Orbea H. (1994). MHC Class II Hierarchy of Superantigen Presentation Predicts Efficiency of Infection with Mouse Mammary Tumor Virus. Int. Immunol..

[64] Kobayashi M., Takaori-Kondo A., Miyauchi Y., Iwai K., Uchiyama T. (2005). Ubiquitination of APOBEC3G by an HIV-1 Vif-Cullin5-Elongin B-Elongin C Complex Is Essential for Vif Function. J. Biol. Chem..

[65] Liu B., Sarkis P.T.N., Luo K., Yu Y., Yu X.-F. (2005). Regulation of Apobec3F and Human Immunodeficiency Virus Type 1 Vif by Vif-Cul5-ElonB/C E3 Ubiquitin Ligase. J. Virol..

[66] Abudu A., Takaori-Kondo A., Izumi T., Shirakawa K., Kobayashi M., Sasada A., Fukunaga K., Uchiyama T. (2006). Murine Retrovirus Escapes from Murine APOBEC3 via Two Distinct Novel Mechanisms. Curr. Biol..

[67] Santiago M.L., Montano M., Benitez R., Messer R.J., Yonemoto W., Chesebro B., Hasenkrug K.J., Greene W.C. (2008). Apobec3 Encodes Rfv3, a Gene Influencing Neutralizing Antibody Control of Retrovirus Infection. Science.

[68] Browne E.P., Littman D.R. (2008). Species-Specific Restriction of Apobec3-Mediated Hypermutation. J. Virol..

[69] Gourzi P., Leonova T., Papavasiliou F.N. (2006). A Role for Activation-Induced Cytidine Deaminase in the Host Response against a Transforming Retrovirus. Immunity.

[70] Ferreira B.L., Ferreira É.R., de Brito M.V., Salu B.R., Oliva M.L.V., Mortara R.A., Orikaza C.M. (2018). BALB/c and C57BL/6 Mice Cytokine Responses to Trypanosoma Cruzi Infection Are Independent of Parasite Strain Infectivity. Front. Microbiol..

[71] Watanabe H., Numata K., Ito T., Takagi K., Matsukawa A. (2004). INNATE IMMUNE RESPONSE IN TH1- AND TH2-DOMINANT MOUSE STRAINS. Shock.

[72] Takeda E., Tsuji-Kawahara S., Sakamoto M., Langlois M.-A., Neuberger M.S., Rada C., Miyazawa M. (2008). Mouse APOBEC3 Restricts Friend Leukemia Virus Infection and Pathogenesis In Vivo. J. Virol..

[73] Sanville B., Dolan M.A., Wollenberg K., Yan Y., Martin C., Yeung M.L., Strebel K., Buckler-White A., Kozak C.A. (2010). Adaptive Evolution of Mus Apobec3 Includes Retroviral Insertion and Positive Selection at Two Clusters of Residues Flanking the Substrate Groove. PLoS Pathog..

[74] Vogt M., Haggblom C., Swift S., Haas M. (1985). Envelope Gene and Long Terminal Repeat Determine the Different Biological Properties of Rauscher, Friend, and Moloney Mink Cell Focus-Inducing Viruses. J. Virol..

[75] Clark S.P., Kaufhold R., Chan A., Mak T.W. (1985). Comparison of the Transcriptional Properties of the Friend and Moloney Retrovirus Long Terminal Repeats: Importance of Tandem Duplications and of the Core Enhancer Sequence. Virology.

[76] Rosen C.A., Haseltine W.A., Lenz J., Ruprecht R., Cloyd M.W. (1985). Tissue Selectivity of Murine Leukemia Virus Infection Is Determined by Long Terminal Repeat Sequences. J. Virol..

[77] Yoshimura F.K., Davison B., Chaffin K. (1985). Murine Leukemia Virus Long Terminal Repeat Sequences Can Enhance Gene Activity in a Cell-Type-Specific Manner. Mol. Cell Biol..

[78] Hakata Y., Landau N.R. (2006). Reversed Functional Organization of Mouse and Human APOBEC3 Cytidine Deaminase Domains. J. Biol. Chem..

[79] Boi S., Kolokithas A., Shepard J., Linwood R., Rosenke K., Van Dis E., Malik F., Evans L.H. (2014). Incorporation of Mouse APOBEC3 into Murine Leukemia Virus Virions Decreases the Activity and Fidelity of Reverse Transcriptase. J. Virol..

[80] Rulli S.J., Mirro J., Hill S.A., Lloyd P., Gorelick R.J., Coffin J.M., Derse D., Rein A. (2008). Interactions of Murine APOBEC3 and Human APOBEC3G with Murine Leukemia Viruses. J. Virol..

[81] MacMillan A.L., Kohli R.M., Ross S.R. (2013). APOBEC3 Inhibition of Mouse Mammary Tumor Virus Infection: The Role of Cytidine Deamination versus Inhibition of Reverse Transcription. J. Virol..

[82] Hagen B., Kraase M., Indikova I., Indik S. (2019). A High Rate of Polymerization during Synthesis of Mouse Mammary Tumor Virus DNA Alleviates Hypermutation by APOBEC3 Proteins. PLoS Pathog..

[83] Manjunath L., Oh S., Ortega P., Bouin A., Bournique E., Sanchez A., Martensen P.M., Auerbach A.A., Becker J.T., Seldin M. (2023). APOBEC3B Drives PKR-Mediated Translation Shutdown and Protects Stress Granules in Response to Viral Infection. Nat. Commun..

[84] Gallois-Montbrun S., Kramer B., Swanson C.M., Byers H., Lynham S., Ward M., Malim M.H. (2007). Antiviral Protein APOBEC3G Localizes to Ribonucleoprotein Complexes Found in P Bodies and Stress Granules. J. Virol..

[85] Kozak S.L., Marin M., Rose K.M., Bystrom C., Kabat D. (2006). The Anti-HIV-1 Editing Enzyme APOBEC3G Binds HIV-1 RNA and Messenger RNAs That Shuttle between Polysomes and Stress Granules. J. Biol. Chem..

[86] Tauber D., Tauber G., Khong A., Van Treeck B., Pelletier J., Parker R. (2020). Modulation of RNA Condensation by the DEAD-Box Protein eIF4A. Cell.

[87] Cui Q., Liu Z., Bai G. (2024). Friend or Foe: The Role of Stress Granule in Neurodegenerative Disease. Neuron.

[88] Pecori R., Di Giorgio S., Paulo Lorenzo J., Nina Papavasiliou F. (2022). Functions and Consequences of AID/APOBEC-Mediated DNA and RNA Deamination. Nat. Rev. Genet..

[89] Walter P., Ron D. (2011). The Unfolded Protein Response: From Stress Pathway to Homeostatic Regulation. Science.

[90] Wang M., Kaufman R.J. (2016). Protein Misfolding in the Endoplasmic Reticulum as a Conduit to Human Disease. Nature.

[91] Foo J., Bellot G., Pervaiz S., Alonso S. (2022). Mitochondria-Mediated Oxidative Stress during Viral Infection. Trends Microbiol..

[92] Gomes M.T.R., Guimarães E.S., Oliveira S.C. (2024). ZBP1 Senses Brucella Abortus DNA Triggering Type I Interferon Signaling Pathway and Unfolded Protein Response Activation. Front. Immunol..

[93] Häsler J., Rada C., Neuberger M.S. (2011). Cytoplasmic Activation-Induced Cytidine Deaminase (AID) Exists in Stoichiometric Complex with Translation Elongation Factor 1α (eEF1A). Proc. Natl. Acad. Sci. USA.

[94] Methot S.P., Litzler L.C., Trajtenberg F., Zahn A., Robert F., Pelletier J., Buschiazzo A., Magor B.G., Di Noia J.M. (2015). Consecutive Interactions with HSP90 and eEF1A Underlie a Functional Maturation and Storage Pathway of AID in the Cytoplasm. J. Exp. Med..

[95] Orthwein A., Patenaude A.-M., Affar E.B., Lamarre A., Young J.C., Di Noia J.M. (2010). Regulation of Activation-Induced Deaminase Stability and Antibody Gene Diversification by Hsp90. J. Exp. Med..

[96] Mediani L., Antoniani F., Galli V., Vinet J., Carrà A.D., Bigi I., Tripathy V., Tiago T., Cimino M., Leo G. (2021). Hsp90-Mediated Regulation of DYRK3 Couples Stress Granule Disassembly and Growth via mTORC1 Signaling. EMBO Rep..

[97] Briggs S., Blomqvist E.K., Cuellar A., Correa D., Burke J.M. (2025). Condensation of Human OAS Proteins Initiates Diverse Antiviral Activities in Response to West Nile Virus. Genes. Dev..

[98] Langereis M.A., Feng Q., van Kuppeveld F.J. (2013). MDA5 Localizes to Stress Granules, but This Localization Is Not Required for the Induction of Type I Interferon. J. Virol..

[99] Loucas G., Locker N., Parker R. (2025). Nucleic Acid-Protein Condensates in Innate Immunity. Mol. Cell.

[100] Reineke L.C., Lloyd R.E. (2013). Diversion of Stress Granules and P-Bodies during Viral Infection. Virology.

[101] Kim K., Calabrese P., Wang S., Qin C., Rao Y., Feng P., Chen X.S. (2022). The Roles of APOBEC-Mediated RNA Editing in SARS-CoV-2 Mutations, Replication and Fitness. Sci. Rep..

[102] Di Giorgio S., Martignano F., Torcia M.G., Mattiuz G., Conticello S.G. (2020). Evidence for Host-Dependent RNA Editing in the Transcriptome of SARS-CoV-2. Sci. Adv..

[103] De Maio N., Walker C.R., Turakhia Y., Lanfear R., Corbett-Detig R., Goldman N. (2021). Mutation Rates and Selection on Synonymous Mutations in SARS-CoV-2. Genome Biol. Evol..

[104] Ratcliff J., Simmonds P. (2021). Potential APOBEC-Mediated RNA Editing of the Genomes of SARS-CoV-2 and Other Coronaviruses and Its Impact on Their Longer Term Evolution. Virology.

[105] Li Z., Luo L., Ju X., Huang S., Lei L., Yu Y., Liu J., Zhang P., Chi T., Ma P. (2024). Viral N Protein Hijacks Deaminase-Containing RNA Granules to Enhance SARS-CoV-2 Mutagenesis. EMBO J..

[106] Derse D., Hill S.A., Princler G., Lloyd P., Heidecker G. (2007). Resistance of Human T Cell Leukemia Virus Type 1 to APOBEC3G Restriction Is Mediated by Elements in Nucleocapsid. Proc. Natl. Acad. Sci. USA.

[107] Shichijo T., Yasunaga J.I., Sato K., Nosaka K., Toyoda K., Watanabe M., Zhang W., Koyanagi Y., Murphy E.L., Bruhn R.L. (2024). Vulnerability to APOBEC3G Linked to the Pathogenicity of Deltaretroviruses. Proc. Natl. Acad. Sci. USA.

[108] Van Norden M., Falls Z., Mandloi S., Segal B.H., Baysal B.E., Samudrala R., Elkin P.L. (2024). The Implications of APOBEC3-Mediated C-to-U RNA Editing for Human Disease. Commun. Biol..

[109] Bishop K.N., Holmes R.K., Sheehy A.M., Malim M.H. (2004). APOBEC-Mediated Editing of Viral RNA. Science.

[110] Teng B., Burant C.F., Davidson N.O. (1993). Molecular Cloning of an Apolipoprotein B Messenger RNA Editing Protein. Science.

